# Convergence of Nanotechnology and Machine Learning: The State of the Art, Challenges, and Perspectives

**DOI:** 10.3390/ijms252212368

**Published:** 2024-11-18

**Authors:** Arnav Tripathy, Akshata Y. Patne, Subhra Mohapatra, Shyam S. Mohapatra

**Affiliations:** 1 Center for Research and Education in Nanobioengineering, Department of Internal Medicine, Morsani College of Medicine, University of South Florida, Tampa, FL 33612, USA; arnav107@usf.edu (A.T.); apatne@usf.edu (A.Y.P.); 2 Graduate Programs, Taneja College of Pharmacy, MDC30, 12908 USF Health Drive, Tampa, FL 33612, USA; 3 Department of Molecular Medicine, Morsani College of Medicine, University of South Florida, Tampa, FL 33612, USA; 4 Research Service, James A. Haley Veterans Hospital, Tampa, FL 33612, USA

**Keywords:** machine learning, deep learning, nanotechnology, cross-collaboration, coevolution, nanoscale, neuromorphic computing

## Abstract

Nanotechnology and machine learning (ML) are rapidly emerging fields with numerous real-world applications in medicine, materials science, computer engineering, and data processing. ML enhances nanotechnology by facilitating the processing of dataset in nanomaterial synthesis, characterization, and optimization of nanoscale properties. Conversely, nanotechnology improves the speed and efficiency of computing power, which is crucial for ML algorithms. Although the capabilities of nanotechnology and ML are still in their infancy, a review of the research literature provides insights into the exciting frontiers of these fields and suggests that their integration can be transformative. Future research directions include developing tools for manipulating nanomaterials and ensuring ethical and unbiased data collection for ML models. This review emphasizes the importance of the coevolution of these technologies and their mutual reinforcement to advance scientific and societal goals.

## 1. Introduction

Nanotechnology is the study and manipulation of materials on the nanoscale, which is defined relative to the nanometer. Working at the nanoscale allows researchers to manipulate the fundamental building blocks of matter to reverse-engineer solutions for drug delivery and agriculture, applying innovations that were previously impossible due to a lack of precision on the macroscale. One of the major contributions of nanotechnology is in the field of microelectronics and semiconductors, including developments like silicon transistors since 1954. These nanoscale components have revolutionized the computing industry and paved the way for advancements in computing power and efficiency.

Machine learning (ML), on the other hand, is an emerging field that derives from artificial intelligence (AI), an idea that began with Alan Turing in the 1940s and accompanied the computer revolution in the last four decades. ML involves the study and training of various algorithms to analyze large datasets, identify patterns, and make independent predictions [1,2]. Through several trials, a user improves the set of algorithms, known as a model, to make more accurate predictions [3]. The discovery of carbon nanomaterials including carbon nanotubes (CNTs) graphene in the early 2000s, provided impetus for early AI algorithms for image recognition, self-driving cars, and the game of checkers [2].

**Figure 1:**
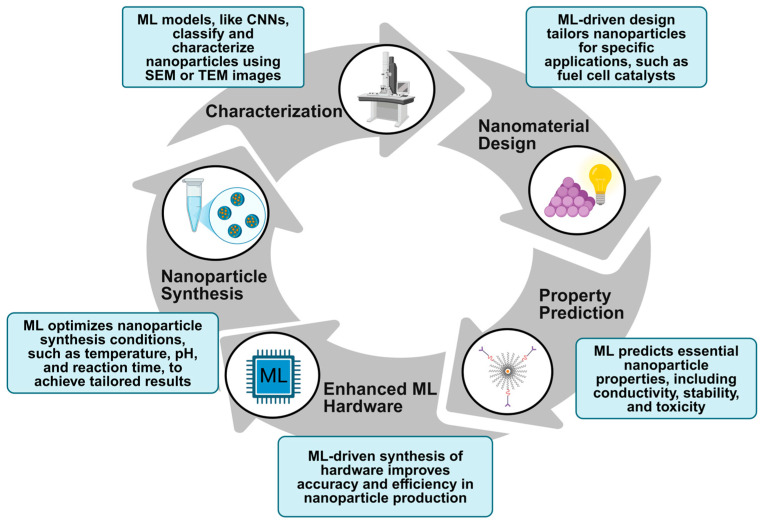
A cartoon depicting convergence and collaboration between ML and nanotechnology.

These two fields—nanotechnology and ML—are increasingly being used in conjunction to address modern technological and scientific challenges, leveraging the strengths of each to advance both. As depicted schematically in Figure 1, ML can help automate some of the aspects of discovering and synthesizing nanoparticles using trained models, accelerating progress in the field. A study conducted in 2021 demonstrated the potential of ML for material discovery by predicting suitable cathode materials for rechargeable zinc batteries, choosing 80 candidates from 130,000 possible materials, 70 of which had never been experimentally tested before [4,5]. Another example of ML’s impact in nanotechnology includes the use of convolutional neural networks (CNNs) to analyze scanning electron microscopy (SEM) images of nanostructures, achieving greater than 95% accuracy in nanoparticle classification [6]. This level of efficiency significantly reduced the time and labor needed for characterization, making it possible to conduct large-scale studies with improved speed and accuracy. These examples underscore ML’s transformative role in discovering nanoparticles with desired properties and automating labor-intensive processes in nanotechnology [7]. Additionally, nanochips can increase the power and efficiency of modern computers, potentially reducing the time and cost of training complex ML models [8].

This review will focus on two aspects of the intersection between nanotechnology and ML: (1) how ML impacts nanotechnology, and (2) how nanotechnology fosters and promotes advances in ML. We aim to highlight the significance of cross-collaboration between these two fields, which can enhance computing capabilities, optimize model training, and accelerate research in nanotechnology and the development of nanomachines. A better understanding of this cross-collaboration is expected to improve the power of computers to optimize model training and speed up research in nanotechnology and direct nanomachines in the future. This review also discusses the challenges in this intersection and future directions.

### 1.1. Synthesis of Nanoparticles

**Figure 2:**
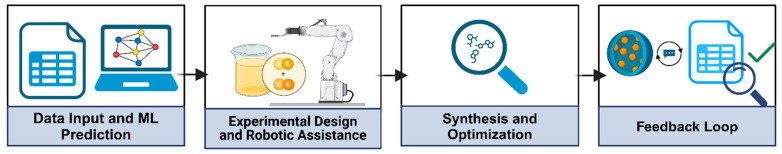
Intelligent automation of nanoparticle synthesis using ML.

One of the largest challenges for the commercial deployment of nanoparticles is the difficulty of effectively processing and manufacturing them. Advances in nanotechnology are often theoretical, with production being a trial-and-error process that is neither cost- nor time-effective. However, cutting-edge ML algorithms are being explored to fully automate the synthesis of nanoparticles, devising optimal synthesis parameters and guiding robotic systems to execute the process efficiently. This enables mass production in a shorter time frame, as described in Figure 2 [9]. ML models predict synthesis conditions such as temperature, pH, and reactant concentration while robotic systems execute these optimized parameters, minimizing human error and speeding up the experimental workflow [10]. This intelligent integration of ML and robotics has the potential to revolutionize nanomaterial production, allowing for more consistent quality and scalability compared with traditional trial-and-error methods [5,11].

The creation of nanomaterials depends strongly on various chemical properties or parameters that the machine accepts, such as temperature, volume, and mass. A model called WANDA (Workstation for Automated Nanomaterials Discovery and Analysis) can map many of these parameters to create a process for synthesis, while a mobile robotic chemist can perform many of the tasks associated with a given process [11]. A major advantage of the system is that it minimizes much of the human error associated with chemistry and works at an unprecedented rate. While the ordinary procedure would require searching “an exhaustive set of synthesis parameters” to achieve the desired structure, followed by hundreds of experimental syntheses, WANDA provides automation that directly searches the synthesis parameter space, optimizing time requirements based on the nanoparticle set [12]. Utilizing ML algorithms with robotic components can potentially revolutionize nanotechnology, bringing nanomaterials to a commercial scale [5,11]. For example, ML could aid in antimicrobial peptide (AMP) discovery. AMPs are found in microorganisms and are used most notably as bactericides. However, they are primarily cationic (positively charged), thus prompting blood-cell rupture, poor bioavailability, and large-scale degradation [13]. With the use of ML and nanotechnology, researchers can discover less volatile, synthetic AMPs that kill bacteria without damaging healthy cells. ML specifically aids in the easy recognition of peptides that are 8–12 amino acids long, increasing sequestration (the storage of carbon), thus leading to better efficiency and more yield [13]. With the development of these new AMPs using ML, researchers can better combat drug-resistant microbes in the body while overcoming traditional challenges with AMPs such as instability and toxicity [14].

Interdisciplinary cooperation, particularly the integration of ML, can optimize production processes and enhance the precision of nanomaterial synthesis, thereby addressing scalability issues more effectively. A recent study highlighted that ML can optimize chemical vapor deposition (CVD) parameters, leading to a 20% increase in the production efficiency of CNTs [15]. Interdisciplinary methods, such as using ML to analyze large datasets of nanoparticle properties, can significantly reduce the time and cost associated with experimental trials [16]. This synergy not only improves efficiency but also opens new avenues for innovation in nanotechnology.

### 1.2. Nanoscale Characterization

**Figure 3:**
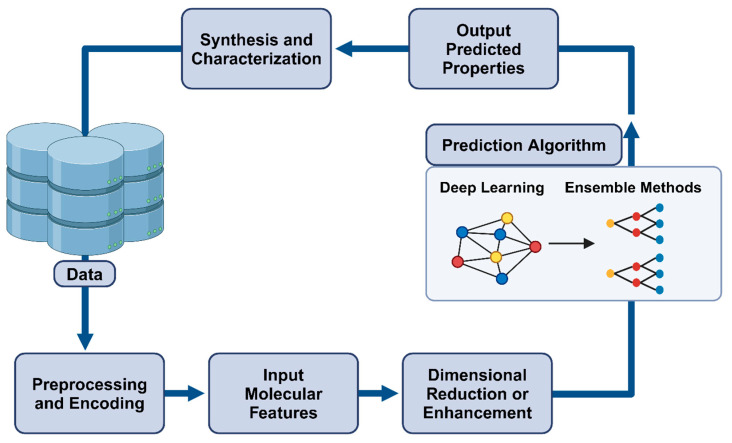
Visualization of the nanostructures examined in the study (scale between 1 and 200 μm) [6,17].

ML excels at drawing meaningful conclusions from incredibly large sets of data, a skill particularly useful in the scientific field. With the vast amounts of visual data scientists need to understand the nanoscale, ML algorithms serve to quickly characterize various structures and patterns with high degrees of accuracy. In a study measuring the capabilities of AI in analyzing scanning electron microscope (SEM) images, advanced neural networks such as Inception-v3, Inception-v4, and ResNet were able to correctly classify 90% of nearly 20,000 images from a training set into one of 10 categories of nanomaterials: tips, particles, patterned surfaces, MEMS devices and electrodes, nanowires, porous sponges, biological, powder, films/coated surfaces, and fibers (Figure 3) [6,17,18].

In a data-heavy field such as nanotechnology, the organization of data (which usually come in the form of images from high-precision SEMs) is critical for other scientists to use in future experiments. As with the synthesis of nanoparticles, the automated classification of images is a powerful tool that cuts down on the time needed to run an experiment [19]. Instead of analyzing images by hand to determine the presence of nanoparticles, often difficult due to low quality images, an ML approach can not only detect these nanoparticles but improve its accuracy by a factor of 3% when trained with synthetic images, as evidenced by a 50-layer mask scoring convoluted neural network trained in 2022 [20]. As ML technology continues to advance, nanomaterials will be analyzed at a faster and more accurate rate than before, catalyzing the engineering process [16].

Also, ML is currently being used to recognize abnormal regularities in X-ray spectroscopy [21]. It was fed data from molecular dynamics simulations, which studied X-ray absorption spectra, and then the researchers extracted partial radial distribution functions, revealing the chemical structure of nanocrystals [22]. ML has also enabled liquid biopsy analysis and has been used in the large-scale manufacturing of nanoscale devices to streamline efficiency and get more of the important products to market [23]. Thus, ML is becoming the forefront for nanoscale characterizations via process flows, predictive metrology, and yield analysis.

### 1.3. Predicting Nanomaterial Properties

ML has appeared as a powerful tool for predicting nanoparticle properties by learning from experimental or simulated data. Three ML algorithms are key to nanoparticle property prediction. First, the Support Vector Machines (SVMs), which are versatile supervised learning algorithms that excel in classification and regression tasks, identify hyperplanes in data to classify or predict properties with high accuracy and apply them in gene classification [24]. SVMs are highly effective supervised learning algorithms that excel in both classification and regression tasks, particularly in the realm of nanocomposite nanomaterials [25]. By adeptly identifying hyperplanes within datasets, SVMs can accurately predict critical properties such as Young’s modulus and tensile strength, often surpassing the performance of conventional regression models [26]. This capability significantly enhances the design process of nanocomposites, enabling researchers to customize mechanical properties for targeted applications while ensuring robust generalization across various datasets. The integration of ML, especially SVMs, has thus been pivotal in advancing the development of nanocomposite materials [27].

Second, random forests are ensemble learning models that include multiple decision trees, trained by randomly sampling data and features, yielding predictions based on the averaged decisions of individual trees [28]. This ML technique can predict the toxicity of mixtures of nanometal oxides and heavy metals. Random forest (RF) models utilize ensemble learning by combining multiple decision trees, significantly improving predictive accuracy and robustness in assessing nanomaterial toxicity [29]. A study by Li et al. (2020) demonstrated that RF models effectively predicted the cytotoxicity of metal oxide nanoparticles with varying compositions. These findings underscore the potential of RF models in guiding the design of safer nanomaterials for biomedical and environmental applications, effectively addressing the complexities associated with nanoparticle toxicity [30].

Neural networks, inspired by the human brain, are versatile algorithms that can be employed in supervised, unsupervised, and reinforcement learning contexts. These networks enable a broad range of applications, including classification and regression. Comprising interconnected nodes for data processing, neural networks can be used in drug testing, optimization, pharmacokinetics, in vitro and in vivo correlations, diagnostic applications, genomics, proteomics, drug design, drug interactions, and potentially many other areas of healthcare [31]. Neural networks can not only classify nanomaterials by shape but also predict the properties of specific nanomaterials by utilizing large chemistry databases and understanding molecular interactions at the nanoscale [32]. For example, a recent report demonstrated a model that successfully predicted the properties of combinations of four, five, and six elements in 18 out of 19 cases without prior knowledge of those materials [33]. This model utilizes a “Megalibrary” that houses billions of different types of nanoparticles and potential combinations for all 118 elements, predicting the structures of nanomaterials scientists have not even created yet [34].

As ML systems advance, new architectures have been created that can predict and discover properties of more complex molecules, such as peptides [35]. With one of the most important parts of nanotechnology being material synthesis, ML can now predict direct applications of nanomaterials, or conversely, suggest nanomaterials that solve a particular problem point (such as treating cancer) [36]. Combined with the virtual chemist and the data-analysis tool WANDA, ML algorithms can significantly enhance the nanoengineering process. Another example is the use of a novel ML approach for predicting nanoparticles’ radial distribution function (RDF) within polymer nanocomposites (PNCs) based on PNC composition, known as the Nanonet framework [37]. RDF measures nanoparticle spatial distribution and significantly affects material properties. NanoNET employs unsupervised dimensionality reduction to encode RDF into a lower-dimensional latent space. A supervised regression model correlates PNC composition with the latent space, enabling the prediction of RDF for any composition [38]. NanoNET was trained and validated using coarse-grained molecular dynamics simulations of PNCs with diverse composition parameters. It proved accurate RDF predictions for unknown PNCs, including compositions absent from the training dataset [38]. Polymer nanocomposites, composed of a polymer matrix and nanofillers, offer exceptional multifunctional properties [27]. Achieving a uniform nanoparticle dispersion within the polymer matrix is crucial, and ML was able to predict nanocomposite properties based on composition and processing parameters, aiding design and optimization.

Another example of ML nanomaterial property prediction pertains to cancer treatment with a real-world impact [39]. Nanotechnology enables cancer treatment using combinations of chemotherapy, radiotherapy, and immunotherapy with MRI-sensitive nanoparticles that monitor the real-time progress of cancer [40]. The advent of ML allows for the specialized direction and deployment of these nanoparticles through predictive analysis [41]. One study used a deep neural network (DNN) to predict the effectiveness of nanoparticle treatment for various tumors in mice. Using 376 different datasets, the DNN predicted responses to the nanoparticles with higher accuracy than the other ML models such as the ensemble model and SVMs [40,42].

### 1.4. Nanodevice Design

**Figure 4:**
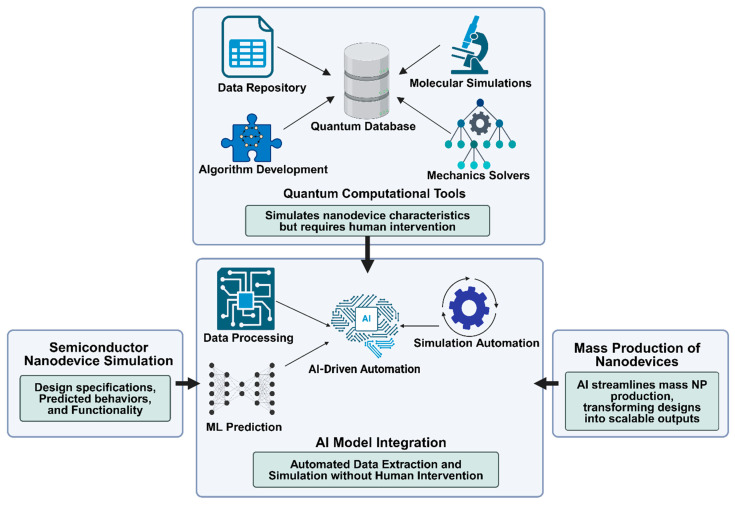
AI-driven design and simulation of nanodevices through quantum databases.

On the nanoscale, devices must follow a set of rules to be functional; often diverging from conventional macro-engineering standards. ML has demonstrated its ability to predict the properties of nanomaterials and can similarly guide the design and invention of nanomachines that traditional computational methods and experiments cannot simulate. Creating a dataset to train a model involves two key tasks: automating the deployment of tools from a quantum database and simulating representations of semiconductor nanodevices without human intervention [43]. This quantum database allows for the simulation of semiconductor nanodevices’ appearance and operation, though it still requires human interaction to utilize its tools. AI is also advancing the mass-scale production of simpler nanodevices, such as perovskites (nanoparticles used in highly efficient LEDs) with the help of catalytic biomolecules [44]. By employing an AI model, researchers can swiftly access data and simulate the specifications and functions of specific nanodevices, as illustrated in Figure 4.

An example of ML’s impact on nanotechnology, particularly in nanomanufacturing, is the construction of tiny devices for various fields [45]. Nanomanufacturing significantly contributes to civil engineering by creating synthetic materials. ML achieve this by analyzing materials with remarkable properties found in nature, such as the strength of spider silk and the adhesive properties of geckos [46]. ML algorithms can leverage these properties to predict new nanomaterials with practical applications, such as in architecture. By using graph neural networks (GNNs), which process graphs as input rather than images, researchers can develop specialized materials tailored to limited structural information about the desired material (the arrangement of points and their connections within the material) [47]. Overall, ML not only aids the development of nanotechnology but also enhances its capacity to address challenges in engineering and medicine.

## 2. Impact of Nanotechnology on ML

While the aspects of ML impacting various aspects of nanotechnology have been extensively researched and reviewed, the converse, i.e., the role of nanotechnology empowering ML, has been less obvious and is more of a recent trend. This is because microelectronics and semiconductors, which utilize nanomaterials and nanosystems, have been traditionally considered separately as a sub-field of nanotechnology [48]. Table 1 summarizes the aspects critical to advancing ML, such as hardware acceleration, data storage, nanomaterials for preprocessing, energy efficiency, and bioinspired computing with examples.

### 2.1. Hardware Acceleration

**Figure 5:**
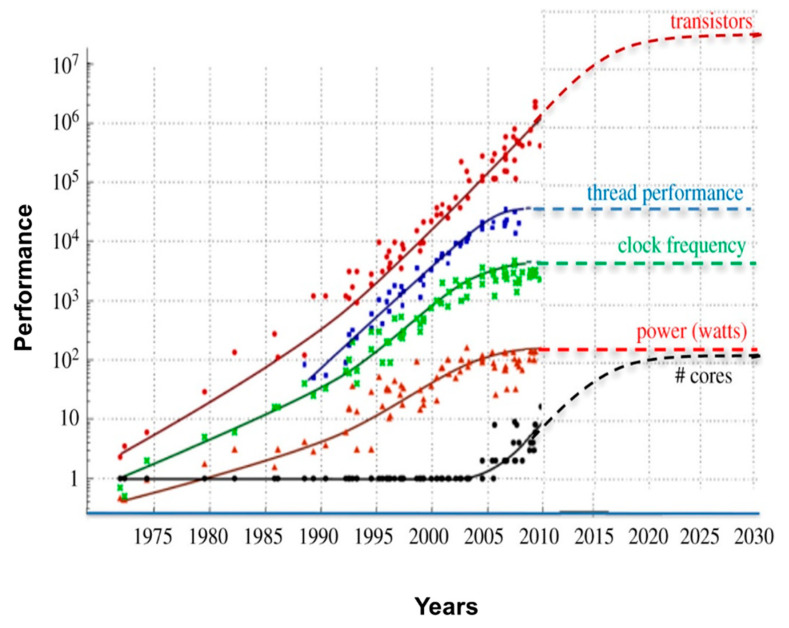
Trends in computing performance metrics from 1975 to 2030, illustrating the challenges in sustaining growth due to physical scaling limitations, such as Dennard scaling and lithography (adapted from 48). The red line illustratets the exponential increase in transistor count, consistent with Moore’s Law. Thread performance, shown by blue line, demonstrates steady gains but begins to plateau as thermal and power constraints limit clock frequency increases, represented by the green line. The red dashed line for power consumption underscores energy efficiency issues as clock speeds reach physical limits. The black dashed line for the number of cores indicates the industry’s shift toward parallelism to overcome these performance bottlenecks.

One of the most fundamental observations in computer engineering, Moore’s Law, posits that the number of transistors on a microchip doubles approximately every two years, driving exponential increases in computing power [49]. Figure 5 illustrates the trends of semiconductor lithography improvements during 1975–2030 and anticipated challenges to maintaining Moore’s Laws and limits due to Dennard scaling in 2004–2025. This reality necessitates exploring alternative materials, such as nanotechnology, to sustain performance growth [50].

With traditional methods for increasing computing power reaching their physical limits, new approaches, including nanotechnology, are essential to drive future performance gains. Notably, nanomaterials like graphene-based transistors and quantum dots operate at higher speeds and with greater efficiency than traditional silicon-based transistors, reducing power consumption and increasing data transfer speeds for ML applications [51].

Silicon-based chips struggle to fit more processing power in smaller areas, so nanomaterials offer a solution to replace or augment silicon chips with highly responsive and conductive nanomaterials such as graphene [52]. Graphene-based transistors and other nano-enabled hardware provide alternative pathways to continue performance improvements beyond the limitations of traditional semiconductor scaling [53]. As nanotechnology improves, researchers aim to increase the scalability of nano-based chips [54].

Taking advantage of the newly developed nanomaterials, ML developers also looking to replace traditional CMOS (complementary metal–oxide–semiconductor) transistors with a new class of devices called memristors. Memristors are nanoscale devices that can change resistance based on electrical impulses, storing and altering data in a memory layer and switching in less than a nanosecond [55,56]. Table 1 highlights how integrating nanotechnology with neuromorphic computing enables the development of nanoscale components that mimic the functionality of biological synapses and neurons, leading to more efficient and powerful computing systems.

Research has shown promise in applications such as pattern recognition, sensor processing, and autonomous navigation, demonstrating the potential of nanotechnology-enabled neuromorphic computing systems [57]. Such solutions provide significant advantages over traditional CMOS transistors by mimicking neural pathways found in the human brain, opening up possibilities for enhanced learning capabilities in AI models and supporting more efficient, brain-like processing for complex tasks [58].

### 2.2. Data Storage

Integrating nanotechnologies with ML can significantly enhance storage densities compared to traditional methods by enabling the storage of larger amounts of data in smaller physical spaces [59]. This optimization of storage solutions can cater to various applications, making storage resources more efficient. For example, using neural networks to predict molecular configurations has improved storage efficiency in molecular memory applications. Additionally, random forests and support vector machines (SVMs) have been applied to optimize nanomaterial synthesis, reducing power constraints while maximizing density for scalable applications [60].

Molecular memory is a revolutionary storage technology that utilizes individual molecules as storage units [61]. It encodes bits of data within the complex structural configurations of molecules, leveraging their unique intricacies and high stability to facilitate precise, high-density data storage and retrieval with exceptional accuracy and efficiency [62]. This innovative technology offers ultra-compact data storage potential for consumer electronics, high-density archival solutions for large data centers, and bio-compatible storage for medical applications [63].

**Figure 6:**
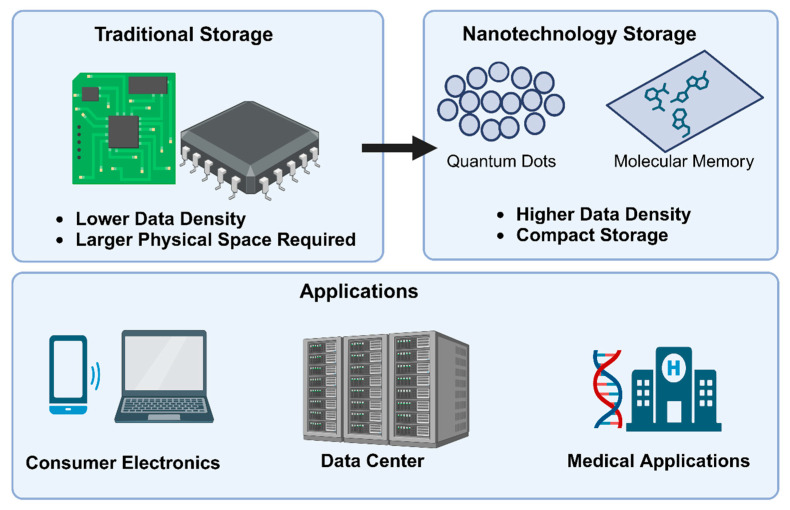
A cartoon of graphene quantum dots (GQDs) and their composites being applied to energy storage devices such as supercapacitors, lithium-ion batteries, solar cells, and fuel cells.

Quantum dot storage is a nanotechnology-driven approach to data storage that utilizes semiconductor nanoparticles at the nanoscale (Figure 6), leveraging quantum mechanics to store and manipulate information at the atomic level. This allows for precise control over electronic states, enhancing data storage density and retrieval speed [64]. Quantum dot storage offers significant advantages in terms of compactness and retrieval efficiency due to their unique electronic properties [65]. Applications range from high-speed, compact storage solutions for consumer devices to integration with quantum computing for advanced data processing [66].

Memristors, for example, have the unique ability to store data within a layer depending on the electrical impulse they receive. These memristors are thin nanowires arranged in a crossbar configuration (i.e., parallel and perpendicular layers), where each intersection functions as a memory cell [67]. By reducing the diameter of these nanowires, it is possible to increase memory density within smaller chips. For instance, 6 nm diameter nanodots or spheres made from nickel and magnesium oxide can store 1 bit per nanodot, packing up to 10 trillion bits (1250 gigabytes) within a single square-inch chip [68]. Enhanced memory capabilities directly improve the cost and effectiveness of training AI models, as large, clunky computers can be replaced by smaller, faster devices that store the same amount of information for large datasets [69]. Some recent examples of the memristors are summarized in Table 2.

### 2.3. Nanomaterials for Data Preprocessing

In the age of modern ML models, image analysis has emerged as a critical asset for generating predictions based on visual inputs. Here, too, nanoengineering enhances the image processing method by addressing latency issues in traditional architectures, where the image sensor and image processor are separated from one another. Nanomaterial-based synaptic optoelectronic devices offer a promising solution by bridging the gap in a way that mimics the human eye, with neural-like pathways connecting the front end to the back end [72].

Neuromorphic computing, an approach to computer engineering that resembles the human brain, can enhance ML models by overcoming technical limitations in processing images [73]. By using transistors that function like neural connections (synapses), models can better replicate the efficiency of the human visual system to process images, creating more accurate facial recognition systems and similar programs [74]. Current artificial synapses are limited, but using nanomaterials such as zinc oxide (ZnO) or nanodots can better mimic the “forgetting behavior” in humans, which is an integral part of learning [75].

Several nanomaterials being considered for use as optoelectronic devices include 2D materials such as graphene and nanoparticles similar to the previously discussed nanodots [76]. The high sensitivity and power of nanomaterials in sensing image input have already been used in ML datasets. For example, indicators for tuberculosis are fed to nanosensors that categorize and compartmentalize information, mitigating the issue of feeding large datasets to ML algorithms. This approach, using a model known as the convoluted neural network (CNN) [77], not only helps transfer image data to the back end of ML architectures but also preprocesses data for the algorithms, expediting their role in disease diagnosis.

### 2.4. Energy Efficiency

One of the largest concerns in ML research is the high energy consumption required for training and deployment. Since models use innumerable data points for generating predictions, multiple training sessions consume resources at an alarming rate, doubling every 3.4 months [78].

Integrating of nanotechnologies can enhance energy efficiencies in data storage and processing systems by reducing power consumption through optimized nanoscale components. This contributes to more sustainable computing solutions and aligns with the increasing focus on energy-efficient technologies [79]. Furthermore, the integration of nanotechnologies with ML can improve the performance and functionality of data storage and processing systems. This can be achieved through faster data access speeds, improved data security measures, and potential integration with other emerging technologies such as quantum computing and AI [80]. These advancements can pave the way for more efficient and secure data handling, ultimately enhancing the overall functionality of computational systems.

One of the most promising nanomaterials, CNTs, can make chips much more energy efficient and thus decrease energy consumption for computers. CNT transistors are so thin that they require a fraction of the energy to operate compared to normal silicon transistors, meaning they still perform the same function while saving power [81]. A prototype nanotube processor has been reported to save 10 times as much energy as similarly sized silicon chips [82]. Once researchers manage to scale it for commercial use, nanotube processors can significantly reduce the massive energy requirements of neural models [83]. Nanomaterials are already being used in minimal-energy AI devices such as smartwatches, with sensors that use 100 times less energy than current technologies [84]. Just as nanotechnology compresses more power into a smaller space, it also retains power without consuming as much energy.

### 2.5. Bio-Inspired Computing

**Figure 7:**
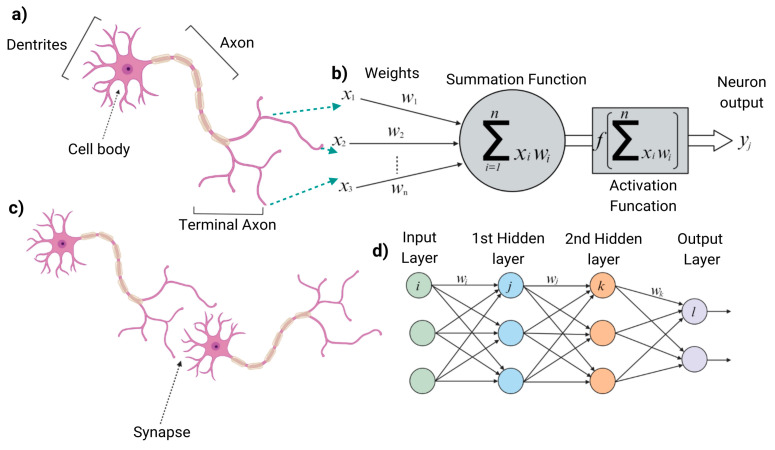
Representation of Spiking Neural Networks (SNNs) and Biological Neuron Analogies. This figure illustrates how SNNs mimic biological neural systems. Part (**a**) shows the structure of a biological neuron with dendrites, a cell body, and axon. Part (**b**) demonstrates the summation and activation functions, paralleling the way a neuron processes input. Part (**c**) shows a synapse, while part (**d**) depicts an SNN with multiple layers (input, hidden, and output), where inputs are summed and processed through an activation function to transmit signals as “spikes”.

ML models that simulate the learning process have drawn significant inspiration from biological processes in the brain. The term “neural network” is based on the idea that these architectures mimic the nervous systems to retain and store information during the learning process [85]. As mentioned earlier, artificial biological components used in ML, such as synaptic optoelectronic devices, are often constructed on the nanoscale, similar to many real-life cells are. CNTs are used in this context, mixed with molybdenum disulfide to create a special type of transistor that “spikes” while generating voltage, much like neurons [86]. Researchers are keen to combine these spiking transistors with the memristors to create artificial memory cells that transfer information in spikes, potentially revolutionizing neural computing [87]. When spiking neurons were used in ML models, creating a new class of neural networks called spiking neural networks (SNNs), the resulting architecture consumed 176,000 times less power than the same network run on a regular chip (Figure 7) [V Lyashenko, https://cnvrg.io/spiking-neural-networks/, accessed on 10 August 2024]. By replicating processes perfected by nature, spiking transistors not only make ML algorithms more effective but also more energy efficient.

SNNs have been used in structural health monitoring for the continuous detection of potential hazards in large buildings using a complex sensor system [88]. Powering such intensive sensor systems is often challenging, so SNNs have been introduced to improve data transmission efficiency. Essentially, sensors do not have to provide high-quality data directly to research facilities but can pass information to an SNN for processing. This allows most of the energy requirement to fall on the maintenance and transmission of the SNNs, reducing overall power consumption [89]. Neural networks, in conjunction with neuromorphic computing, can act as an artificial “ear” that extracts damage-sensitive features (DSFs) from the sensors and processes them with minimal energy, improving safety and alerting to possible hazards in buildings [90,91]. This auditory sensing capability of SNNs also enhances speech recognition. Compared to traditional CMOS semiconductor networks, SNNs require far less energy to process spatiotemporal data, such as audio [92]. Audio signals from speech are extracted and converted into spikes by an algorithm and a 1D self-organizing map network (a simple type of ML in which complex data are converted into simpler data). This information is then used to train an SNN powered by memristors made of tungsten, magnesium oxide, silicon dioxide, and molybdenum, achieving a 94% speech recognition accuracy in one study [93].

As research into the speech recognition capabilities of SNNs progresses, data from human MRI scans have been used to create better neural networks based on how the human brain reacts to speech [94]. The model makes use of artificial excitatory and inhibitory neurons, which increase and decrease activity at a certain connection, respectively. Finding the balance between these two synapses in neuromorphic computing, specifically a time delay between their responses, can revolutionize speech recognition capabilities in SNNs [95,96]. Overall, spiking neural networks built upon nanotechnological memristors have enormous real-world potential, such as in speech recognition and damage detection in buildings.

## 3. Limitations and Future Directions

### 3.1. Current Issues with Nanotechnology and ML

The integration of nanotechnology and ML faces significant challenges that require interdisciplinary collaboration to overcome. One of the core challenges in nanotechnology is scalability. While ML can optimize parameters to improve synthesis efficiency, scaling up nanomaterial production from laboratory settings to industrial applications remains difficult due to the high precision required in particle size, composition, and morphology [97]. For example, ML algorithms can predict optimal synthesis parameters, such as temperature and reactant concentration, but translating these predictions into consistent, large-scale manufacturing processes is complex [98]. Additionally, producing nanomaterials on a large scale may lead to significant chemical waste and by-products, raising environmental, health, and safety (EHS) concerns [99]. These EHS issues are particularly relevant to nanotechnology, where nanoparticle exposure and disposal can adversely affect ecosystems and human health.

The black-box nature of complex ML models, particularly deep neural networks, presents another challenge for nanotechnology [100]. Model transparency is essential in nanotechnology applications where understanding specific model predictions, such as toxicity or stability of nanomaterials, is crucial for regulatory approval and public trust. Explainable AI (XAI) techniques, which aim to enhance interpretability, are especially valuable in nanotechnology applications to ensure that ML-driven predictions are reliable and actionable [101]. For instance, when ML is used to predict the toxicity of novel nanomaterials, interpretability allows researchers and regulators to understand the basis of these predictions, thereby facilitating safer product development [102].

Furthermore, ML models present challenges related to fragility and bias. This not only includes harming nanomaterial discovery by erroneously providing details based on incomplete training but also, in general, applications that use large language models like GPT-3 [103]. Bias in training data can impact the accuracy of ML predictions in nanomaterial discovery and beyond. Another significant issue is the “black box” nature of complex ML models, particularly deep neural networks, which reduces interpretability and hinders their adoption in critical applications [104]. XAI techniques, aiming to enhance model transparency, are an active area of research [105]. Integrating XAI into nanotechnology-related ML applications can build trust in model predictions and facilitate adoption.

Both fields face unique yet interconnected challenges that require a nuanced approach to ensure effective integration. The production of nanotechnology-enabled hardware, for example, demands highly specialized manufacturing processes to achieve the precision required at the nanoscale [106]. This precision is critical for developing devices such as nanoscale transistors for ML acceleration, where even minor defects can significantly impact performance. Consequently, scalability is often limited by the need for extremely controlled environments, which are challenging and costly to maintain on a large scale [107]. Quality control also presents a substantial challenge, as small inconsistencies in nanomaterial properties can lead to deviations in device performance. Ensuring consistent quality at scale requires continuous improvements in quality assurance methodologies specifically tailored to the unique characteristics of nanomaterials [108]. Another hurdle lies in adapting ML software and algorithms to fully utilize nanotechnology-enabled hardware accelerators. Current ML models are often energy-intensive, posing challenges for integration with energy-efficient nanoscale hardware, particularly in environments where energy constraints are a concern [109]. Optimizing ML models for energy efficiency, therefore, becomes essential as we work toward seamlessly integrating ML capabilities with nanoscale hardware [110].

Ethical concerns are a critical challenge shared by both nanotechnology and ML, underscoring the importance of interdisciplinary approaches to governance [102]. Collaborative efforts between ethicists, nanotechnologists, and ML experts are crucial to developing comprehensive guidelines that ensure the safe and responsible use of these technologies [111]. Specific regulatory frameworks are necessary to prevent unintended consequences, such as environmental damage from nanomaterial waste or the misuse of AI for surveillance [112]. Ethical and environmental considerations must be central to the development and deployment of these technologies.

Recent initiatives, such as green nanotechnology principles and ethical guidelines for AI, emphasize transparency, accountability, and sustainability in applying nanotechnology and AI. Collaboration among scientists, ethicists, policymakers, and the public will be essential to balance innovation with safety, ensuring that nanotechnology and ML are developed responsibly and sustainably [113].

### 3.2. Future Directions

Despite these challenges, ML offers significant opportunities to enhance the advances of nanotechnology. For example, ML can optimize the synthesis parameters (e.g., temperature, pressure, and catalyst type) to reduce production costs of CNTs by predicting the most efficient conditions, streamlining processes like CVD [114]. Additionally, integrating ML in the synthesis of CNTs can automate monitoring and control systems, enhancing the precision of complex synthesis methods, which can lead to improved yield and quality while minimizing human error and operational costs. ML can also be utilized for predictive maintenance of equipment used in CNT production, reducing downtime and maintenance costs by forecasting potential failures through data analysis from machinery, ensuring continuous production and efficiency [115]. This includes screening large nanoparticle libraries to identify candidates with desired properties, designing nanoparticles with specific properties in mind, optimizing nanoparticle synthesis processes for improved yield and quality, accelerating the discovery of new nanoparticles with unprecedented properties and functionalities, and developing inverse design methods to design nanoparticles and polymer nanocomposites with desired properties by optimizing their structural features.

The progress of nanotechnology hinges on interdisciplinary research that unites expertise from various fields. Collaborative efforts can lead to the development of high-precision tools and models that bridge the gap between macro- and nanoscale phenomena, facilitating breakthroughs in nanoengineering. A recent interdisciplinary study successfully used ML to predict the mechanical properties of nanocomposites, which were then validated experimentally with high accuracy [88]. Firstly, scientists must find a way to properly regulate nanomaterials on an atomic level. High-precision microscopes, such as SEMs and AFMs (Atomic-Force Microscopes), have helped visualize nanomachines, but engineers have yet to develop more tools for manipulating them, ensuring that all parts fit together with very little tolerance. Another point of interest is tying nanoengineering to a model of quantum mechanics. Uniting macrophysics with nanophysics has long been a goal for theoretical scientists, but finding a solution can be of high importance to the realm of nanoengineering, allowing researchers to successfully create equations modeling forces and the behavior of nanoscale particles in the construction of machines [116]. In healthcare, the combination of these fields can enable the development of personalized medicine and diagnostics, leading to more targeted and effective treatments. Furthermore, nanotechnology-enabled sensors and ML algorithms can revolutionize environmental monitoring and remediation efforts, helping to address pressing issues such as pollution and climate change.

Meanwhile, AI also has room for improvement beyond energy and time constraints. One key area is reducing bias in the datasets used to train ML models. Researchers often use ubiquitous benchmark datasets to test algorithms and determine their quality. Unfortunately, these datasets can create internal biases in the algorithms that affect their performance on real-world data. Therefore, it is crucial to collect as much real-world data as possible that is specific to a given model, ensuring that this data is collected ethically, especially when training models with medical data [117].

The convergence of nanotechnology and ML also presents opportunities for addressing global challenges. For example, ML-powered nanotechnology can contribute to the development of sustainable energy solutions, such as more efficient solar cells and batteries. Nanotechnology-enabled ML hardware accelerators are experiencing remarkable growth. This growth can be attributed to two key factors. First, as ML becomes increasingly integral to various industries, the demand for efficient and high-performing ML hardware accelerators has grown exponentially [118]. Applications span healthcare, finance, manufacturing, autonomous vehicles, image recognition, and real-time data analysis. Second, the continued development and refinement of nanotechnology-enabled ML hardware accelerators are crucial drivers of market growth. Manufacturers are striving to create more efficient and powerful hardware, constantly pushing the boundaries of what is achievable. The adoption of cloud-based ML services is transforming the landscape by providing businesses access to powerful ML hardware accelerators without the need for heavy investment in dedicated hardware. As a result, ML is becoming more accessible to companies of varying sizes and budgets.

## 4. Conclusions

Nanotechnology and ML provide invaluable assistance to each other’s development, with the potential to revolutionize computing power, energy storage, and medical technology. Through nanotechnology, specialized chips and systems can be created to improve the efficiency of ML models, leading to a new generation of neural networks that mimic the human brain. Meanwhile, ML can facilitate the discovery and synthesis of new nanomaterials using previously acquired knowledge, saving chemical engineers significant time and effort in accelerating the progress of nanotechnology [119].

These two fields, working together, hold the potential to usher in a new age of scientific advancement by automating medical, agricultural, and aeronautical technology with unprecedented power and efficiency. For example, in agriculture, nanotechnology-enabled sensors combined with ML algorithms could monitor soil health and optimize crop yield. In aeronautics, nanomaterials could be used to create lightweight components, and ML could predict maintenance needs, improving safety and efficiency [120]. ML hardware acceleration is entering a new age because of nanotechnology, making ML more accessible, quick, and energy-efficient, with the potential to transform various sectors.

However, significant challenges remain, such as manufacturing complexity and the need for specialized software tools. Recognizing these challenges provides a balanced perspective on the future of these technologies. Despite these obstacles, the advantages in transforming the AI landscape are undeniable. Advances in nanomedicine, in particular, demonstrate the potential of these tools, which will become increasingly critical to the development of ML as nanotechnology advances. The combination of nanotechnology and ML opens new opportunities for addressing challenging issues and enabling previously unimaginable applications.

## Figures and Tables

**Table 1:** Examples of nanotechnology affecting ML approaches, speed, and efficiency.

Parameters	Nanomaterial/ Technology	ML Benefits	Applications
Hardware Acceleration	Graphene-based transistors, quantum dots	Operate at higher speeds and with greater efficiency than traditional silicon-based transistors, reducing power consumption, and increasing speed of data transfer for ML applications.	High-speed ML applications, especially deep learning models and real-time image processing.
Data Storage	Nanowire-based memristors, molecular memory	Enable high-density storage in a small footprint, supporting large datasets required for ML without power constraints.	Compact data storage for ML models, large-scale data centers, neuromorphic computing hardware.
Neuromorphic Computing	Memristors (e.g., nanocrystalline ZnO, TiO_2_)	Mimic synaptic functions, providing faster data processing and enabling ML algorithms to learn like biological neurons.	Pattern recognition, autonomous navigation, sensor data processing for ML and AI applications.
Data Processing	Spintronics, nanosensors	High-speed data access, reduced latency, and energy-efficient processing by leveraging spin properties for faster data retrieval.	ML-based edge computing, real-time environmental monitoring, and health diagnostics.
Energy Efficiency	Graphene supercapacitors, thermoelectric materials	Provide rapid energy discharge and reduce overall power consumption, supporting sustainable and efficient ML operations.	Edge computing devices, energy-limited applications, and high-performance ML hardware.

**Table 2:** Examples of memristors accelerating nanotechnology-inspired ML.

Goal	Innovation	Advantages	Applications	Storage Density	Power Consumption	Reference
To create memory devices with high storage density and low power consumption for neuromorphic computing	Develop novel memristors using MXene composite with nanocrystals to emulate synaptic properties and enhance data density	- High-density data storage	Neuromorphic computing systems	High (e.g., 10 Tb/in^2^)	Very low (<1 mW)	[70]
		- Low power consumption				
		- Tunable gate properties				
		- Integration with existing electronics				
To build scalable parylene-based memristors with improved memory stability for ML models	Optimize Ag nanocomposite in a parylene-based memristor structure for enhanced stability and reduced data loss	- Reduced internal stochasticity	ML hardware, data storage	Moderate (e.g., 5 Tb/in^2^)	Moderate (5 mW)	[71]
		- Improved memory stability for ML applications				
		- Simplified architecture for neural networks				
To create nanocrystalline ZnO-based memristors for compact, high-density storage in AI hardware	Implement ZnO-based memristors with multi-layer nanostructures for improved storage capacity and reliability	- Enhanced endurance and data retention	AI hardware, consumer electronics	High (e.g., 8 Tb/in^2^)	Low (2 mW)	[37]
		- High to low resistance ratio				
		- Suitability for compact AI devices				
		- Potential for replicating short-term synaptic plasticity				

## Data Availability

All other data generated or analyzed during this study are included in this published article.

## Abbreviations

ML	Machine Learning
AI	Artificial Intelligence
WANDA	Workstation for Automated Nanomaterials Discovery and Analysis
AMP	Antimicrobial Peptide
CVD	Chemical Vapor Deposition
CNT	Carbon Nanotube
SEM	Scanning Electron Microscope
SVM	Support Vector Machines
RF	Random Forest
RDF	Radial Distribution Function
PNC	Polymer Nanocomposite
MRI	Magnetic Resonance Imaging
DNN	Deep Neural Network
LED	Light-Emitting Diode
GNN	Graph Neural Networks
CMOS	Complementary Metal-Oxide Semiconductors
CNN	Convoluted Neural Network
SNN	Spiking Neural Network
DSF	Damage Sensitive Features
EHS	Environment, Health, and Safety
